# High-resolution cryo-EM proteasome structures in drug development

**DOI:** 10.1107/S2059798317007021

**Published:** 2017-05-31

**Authors:** Edward P. Morris, Paula C. A. da Fonseca

**Affiliations:** a Division of Structural Biology, The Institute of Cancer Research, Chester Beatty Laboratories, 237 Fulham Road, London SW3 6JB, England; b MRC Laboratory of Molecular Biology, Francis Crick Avenue, Cambridge CB2 0QH, England

**Keywords:** electron microscopy, cryo-EM, single particle, proteasome, inhibitors, drug design, human, *Plasmodium falciparum*, malaria, *icr*3*d*, *icr*3*dpro*

## Abstract

With the recent advances in biological structural electron microscopy (EM), protein structures can now be obtained by cryo-EM and single-particle analysis at resolutions that used to be achievable only by crystallographic or NMR methods. We have explored their application to study protein–ligand inter­actions using the human 20S proteasome, a well established target for cancer therapy that is also being investigated as a target for an increasing range of other medical conditions. The map of a ligand-bound human 20S proteasome served as a proof of principle that cryo-EM is emerging as a realistic approach for more general structural studies of protein–ligand interactions, with the potential benefits of extending such studies to complexes that are unfavourable to other methods and allowing structure determination under conditions that are closer to physiological, preserving ligand specificity towards closely related binding sites. Subsequently, the cryo-EM structure of the *Plasmodium falciparum* 20S proteasome, with a new prototype specific inhibitor bound, revealed the molecular basis for the ligand specificity towards the parasite complex, which provides a framework to guide the development of highly needed new-generation antimalarials. Here, the cryo-EM analysis of the ligand-bound human and *P. falciparum* 20S proteasomes is reviewed, and a complete description of the methods used for structure determination is provided, including the strategy to overcome the bias orientation of the human 20S proteasome on electron-microscope grids and details of the *icr*3*d* software used for three-dimensional reconstruction.

## Abstract 2

High-resolution cryo-EM was used to investigate the structures of inhibitor-bound human and *P. falciparum* 20S proteasomes, revealing the molecular basis for inhibitor specificity that provides a platform for the development of a potential new class of antimalarials. Here, these studies are reviewed and a detailed description of the methods used for structure determination is provided.

## 1. Introduction   

The growing understanding of the intricate mechanisms underlying cellular function, and how these are disrupted in disease, allows the identification of specific molecular targets that can be modulated by chemical compounds with the potential to be developed as next-generation drugs for clinical use. The proteasome is a large protease complex that is essential in all eukaryotes. It not only contributes to overall proteostasis, but also plays a critical role in the highly regulated ATP-dependent degradation of specific ubiquitin-tagged proteins, the removal of which triggers fundamental mechanisms such as cell-cycle progression and apoptosis. The proteasome comprises a proteolytic core, the 20S proteasome, which encloses the proteolytic active sites (Lowe *et al.*, 1995 ▸; Groll *et al.*, 1997 ▸). In eukaryotes, the 20S proteasome is formed by hetero-heptameric rings of homologous α and β subunits arranged as a twofold-symmetric α_(1–7)_β_(1–7)_β_(1–7)_α_(1–7)_ barrel-shaped stack (Groll *et al.*, 1997 ▸; Unno *et al.*, 2002 ▸; Harshbarger *et al.*, 2015 ▸). The active sites of the proteolytic subunits, β1, β2 and β5, are located within the inner cavity of the 20S proteasome and have distinct amino-acid sequence-cleavage specificities, namely caspase-like, trypsin-like and chymotrypsin-like, respectively (Heinemeyer *et al.*, 1997 ▸). Variant forms of the proteasome can be found in higher eukaryotes (Kniepert & Groettrup, 2014 ▸; Dahlmann, 2016 ▸). These include the immunoproteasome, in which the active subunits of the constitutive proteasome are replaced by the interferon γ-induced β1i, β2i and β5i counterparts (Kuckelkorn *et al.*, 1995 ▸; Groettrup *et al.*, 1996 ▸), which are critical for the generation of antigenic peptides for major histocompatibility class I presentation. Other proteasome variants include the thymoproteasome (Murata *et al.*, 2007 ▸), which is found in the thymic cortex, where the β5t subunit variant is expressed and incorporated into the proteasome together with β1i and β2i, and the spermatoproteasome, a mammalian variant in which the constitutive α4 subunit is replaced by testis-specific α4s (Yuan *et al.*, 1996 ▸). The 20S proteasome on its own provides limited access to its active sites. Accordingly, the 20S proteasome has only limited proteolytic activity towards small peptides and disordered proteins (Groll *et al.*, 2000 ▸), and its full activation requires the association of regulatory particles that bind at the outer surfaces of the proteasome α rings. The 19S regulatory particle is the proteasome regulator that recruits fully folded ubiquitinated protein substrates for degradation and proceeds with their ATP-dependent unfolding and translocation towards the proteolytic sites of the 20S proteasome.

The proteasome is a well established target for therapeutic drug development (Kisselev *et al.*, 2012 ▸), including the treatment of cancer (Manasanch & Orlowski, 2017 ▸). Bortezomib was the first proteasome inhibitor to be approved for clinical use against multiple myeloma. Subsequently, two second-generation inhibitors have been approved for the treatment of relapsed or refractory multiple myeloma, namely carfilzomib (Moreau *et al.*, 2012 ▸) and, more recently, ixazomib (Muz *et al.*, 2016 ▸), the first orally administered proteasome inhibitor. Next-generation proteasome inhibitors are being developed for higher efficacy and for the treatment of a wider range of medical conditions. These include compounds aimed at selectively targeting proteasome subtypes, namely constitutive proteasomes or immunoproteasomes, with minimum cross-reactivity in order to minimize off-target toxicity (Huber & Groll, 2012 ▸; Huber *et al.*, 2016 ▸; Xin *et al.*, 2016 ▸). The development of drugs that specifically target the immunoproteasome further extends the therapeutic potential of proteasome inhibitors, including to the treatment of inflammatory dis­orders (Basler *et al.*, 2015 ▸). Below, we discuss the potential of targeting the proteasome against malaria, and the likely extension of this approach to other protozoan infections.

Drug discovery is often guided by the knowledge of the structure of a protein target and of its interactions, at the atomic level, with candidate or prototype ligands. The first crystal structure of a 20S proteasome was obtained for a simpler archaeal complex (Lowe *et al.*, 1995 ▸). Subsequently, eukaryotic 20S proteasome crystal structures were determined for yeast (Groll *et al.*, 1997 ▸), constitutive bovine and human complexes (Unno *et al.*, 2002 ▸; Harshbarger *et al.*, 2015 ▸), and the constitutive proteasomes and immunoproteasomes from mouse (Huber *et al.*, 2012 ▸). Over the years, X-ray crystallo­graphy has been pivotal in providing structural information for the development of proteasome inhibitors as therapeutic agents (Groll & Huber, 2004 ▸; Borissenko & Groll, 2007 ▸). However, the field of biological structural electron microscopy has recently seen an enormous transformation (Kuhlbrandt, 2014 ▸; Vinothkumar & Henderson, 2016 ▸), and high-resolution protein structures can now be obtained using electron cryomicroscopy (cryo-EM) and single-particle analysis. We have explored their applicability to study protein–ligand inter­actions using first the human 20S proteasome core, providing a proof of principle for the potential use of these methods in structure-based drug discovery and development (da Fonseca & Morris, 2015 ▸). Subsequently, a similar approach was used to provide the structural information needed to validate the *Plasmodium falciparum* proteasome as a viable molecular target against malaria and also to guide the improvement of the prototype specific *Plasmodium* proteasome inhibitors tested as potential antimalarials (Li, O’Donoghue *et al.*, 2016 ▸; Li, Bogyo *et al.*, 2016 ▸). Within the context of the proceedings of the Second CCP-EM Spring Symposium, here we review our cryo-EM analysis of ligand-bound human and *P. falciparum* proteasomes, focusing on a detailed description of the methods we used for structure determination, including our strategy to avoid orientation bias of the proteasome on electron-microscope grids and the *icr*3*d* program used for three-dimensional reconstruction.

## 2. Cryo-EM of eukaryotic 20S proteasomes   

The behaviour of 20S proteasomes from higher eukaryotes on electron-microscope grids diverges from that of archaeal proteasomes, which have been used as reliable test samples in the development of cryo-EM and image-processing methods. In this context, structures of archaeal 20S proteasomes have been determined by cryo-EM and single-particle analysis at resolutions of 2.8 Å (Campbell *et al.*, 2015 ▸; Grant & Grigorieff, 2015 ▸) and 2.4 Å (Danev *et al.*, 2017 ▸), the latter by the analysis of images recorded using a phase plate. Such cryo-EM studies take advantage of the high stability and homogeneity of the archaeal complex at high concentrations and under buffer conditions that are highly suitable for the preparation of cryo-EM grids. Furthermore, the lower complexity of the archaeal proteasome, which is formed by homo-heptameric rings of α and β subunits, results in a higher order *D*7 symmetry assembly. This allows a sevenfold increase in internal averaging of the archaeal proteasome subunits compared with that in the *C*2 symmetrical eukaryotic complexes, which greatly facilitates image processing. The cryo-EM analysis of the archaeal 20S proteasome can in principle be performed with a significantly smaller stack of molecular images than is required for the analysis of eukaryotic complexes, making the analysis significantly less computationally demanding. Furthermore, the pseudo-symmetry of the eukaryotic 20S proteasome can lead to image misalignments that are out of register around the pseudo-sevenfold axis, which do not occur with the exact sevenfold symmetry of the archaeal α and β subunit rings. On the other hand, the structural analysis of archaeal complexes cannot provide information on the ligand specificity and selectivity of each of the three distinct proteolytic sites of eukaryotic 20S proteasomes. The selectivity of each of the three distinct proteolytic active sites of the eukaryotic complex, associated with subunits β1, β2 and β5, is dictated by the different amino-acid side chains lining each of the ligand-binding pockets, which differ from those lining the single archaeal active site. Therefore, detailed structural information on each of the eukaryotic active sites is essential to assist drug development.

### 2.1. Preparation of cryo-EM grids of 20S proteasome samples from higher eukaryotes   

Samples of the human and *P. falciparum* 20S proteasome cores were incubated in solution for 1 h at 37°C in the presence of a concentration of ligand optimal for maximal binding while still preserving active-site specificity, as determined by *in vitro* binding assays under similar conditions (da Fonseca & Morris, 2015 ▸; Li, O’Donoghue *et al.*, 2016 ▸). After incubation, the samples were loaded onto electron-microscope grids. We used Quantifoil 1.2/1.3 grids freshly coated with a thin layer of carbon. For the preparation of these grids, thin carbon films were prepared by carbon evaporation onto freshly cleaved mica using an Edwards Auto 306 coating unit. The Quantifoil grids were quickly dipped in acetone for 2–3 s in order to improve their wettability, and immediately placed on filter paper submerged in ultrafiltered water within a Petri dish. The carbon film was floated from the mica surface and the filter paper was raised so that the carbon film was harvested on the surfaces of the grids. After the grids had been rendered hydrophilic by glow discharge, 2 µl of 20S proteasome sample was applied onto the thin carbon for approximately 20 s, the excess solution was removed by blotting and the grids were flash-frozen into vitreous ice using an FEI Vitrobot.

**Figure 1:**
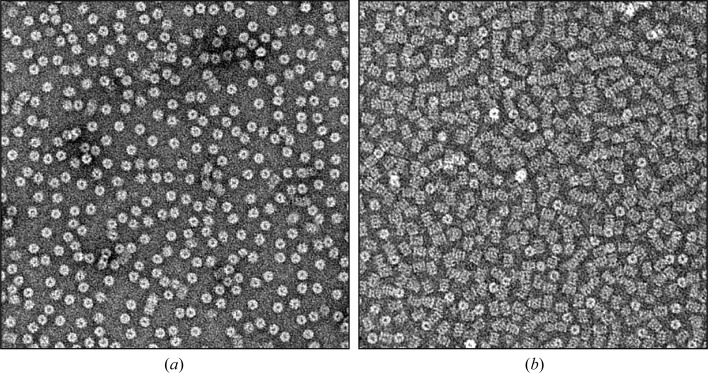
EM images of negatively stained fields of 20S proteasome complexes showing the effect of altering the surface charge of the carbon support film on the orientation of the human 20S proteasome. (*a*) Glow discharge of the EM grid in a partial vacuum of atmospheric air results in a strongly biased proteasome orientation with predominant top views. (*b*) Glow discharge in a partial vacuum containing pentylamine vapour results in a predominance of side views.

In the case of the human 20S proteasome, glow discharge of the electron-microscope grids in a partial vacuum of atmospheric air, at ∼20 Pa for ∼20 s, using an Emitech K950X led to a strongly preferred top-view orientation of the complexes. Under these conditions, the outer surface of the proteasome α-rings preferentially interacts with the carbon film (Fig. 1 ▸ *a*), a behaviour that has previously been observed for other eukaryotic 20S proteasome samples (Baumeister *et al.*, 1988 ▸; Tanaka *et al.*, 1988 ▸). Since data sets with this orientation bias are not suitable for three-dimensional analysis, we investigated modifying the glow-discharge protocol. We found that glow discharge of the grids in the presence of pentylamine (also known as amylamine), at a pressure of ∼50 Pa for ∼20 s, resulted in a radical reorientation of the human proteasome, with >90% of the molecular images corresponding to side views perpendicular to the long axis of the proteasome (Fig. 1 ▸ *b*). Since these side views have a complete and even distribution 360° around the proteasome central axis, they are well suited for an accurate and isotropic three-dimensional reconstruction (da Fonseca & Morris, 2015 ▸). In order to render the carbon surface of the electron-microscope grids hydrophilic and suitable for an adequate orientation of the human proteasome, pentylamine was introduced into the glow-discharge chamber either as 3 × 50 µl drops on a piece of filter paper or as 50 µl in a small open vial. Interestingly, the 20S proteasome from *P. falciparum* showed a different behaviour and yielded a reasonable mixture of top and side views on carbon films glow-discharged in atmospheric air (Li, O’Donoghue *et al.*, 2016 ▸), appearing to be closer to that observed for archaeal proteasomes. Since pentylamine treatment did not seem to affect this distribution, it was not used in the glow discharge of grids prepared for structural analysis of the *Plasmodium* complex.

Glow discharge in the presence of pentylamine has previously been observed to modify the adhesion of macromolecules to carbon films by creating a positively charged surface, in contrast to the negative charge obtained by glow discharge in atmospheric air (Dubochet *et al.*, 1971 ▸; Aebi & Pollard, 1987 ▸). We were able to replicate this change in the orientation of the human 20S proteasome in a number of different glow-discharge units. However, in all cases we observed that significant care was required to maintain the positive charge on the carbon during glow discharge. If the glow discharge was either prolonged significantly beyond 20 s or the vacuum was allowed to increase significantly, the pentylamine effect was reversed and the grids appeared to become negatively charged, with the predominance of top views of the human 20S proteasome returning. This is related to the colour of the glow discharge: in the presence of pentylamine the glow has a characteristic pure blue colour, whereas in air it is violet (Aebi & Pollard, 1987 ▸), and loss of the required positive charge can be monitored by a transition of the colour of the glow from the desired blue to violet. We also observed that the 20S proteasome from *Saccharomyces cerevisiae* shows the same orientation changes with different carbon charges as the human complex. For a more general application in cryo-EM, the sensitivity of the orientation of human and yeast 20S proteasomes to the polarity of the charge at the carbon surface, their ready availability from commercial sources and the ease with which proteasome top and side views can be distinguished make them a useful control for evaluating the effectiveness of glow-discharge procedures of carbon films in the presence of pentylamine. When preparing batches of glow-discharged grids to image other proteins or protein complexes, any particle reorientation may not be as obvious as for the proteasome. Because the charge of the carbon can be easily reversed during glow discharge in the presence of pentyl­amine, a simple way to evaluate it is to load a single grid from a batch of treated grids with human or yeast 20S proteasomes, which can be negatively stained and readily imaged by electron microscopy at room temperature. The orientation of the proteasomes in such a grid provides a control for the efficiency of the pentylamine treatment for all of the grids in the batch glow-discharged at the same time. When the pentylamine effect is successfully achieved, the positive charges at the carbon surfaces are stable for a few hours.

### 2.2. High-resolution cryo-EM data collection   

We have described our strategy to collect high-resolution cryo-EM images of both ligand-bound human and *P. falciparum* 20S proteasome samples (da Fonseca & Morris, 2015 ▸; Li, O’Donoghue *et al.*, 2016 ▸). Briefly, 20S proteasome molecular images were recorded using an FEI Titan Krios electron microscope with a Falcon II direct electron detector. In both cases images were captured as 17 individual frames during a 1 s exposure at a calibrated sampling of 1.04 Å pixel^−1^. All recorded images were inspected for their signal-to-noise ratio and the recovery of isotropic high-resolution information to at least about 4 Å, as evaluated by the recovery of contrast-transfer function modulation in the image power spectra. For the images selected for further analysis the sum of all frames, corresponding to an accumulated dose of about 50 e^−^ Å^−2^, was used for particle picking, taking advantage of the higher signal-to-noise ratio that facilitates the unambiguous identification of molecular images. The subsequent image-processing and three-dimensional refinement procedures were performed using the sum of frames 3–10 of each selected image. The first recorded frames were excluded since beam-induced particle movements are significantly accentuated in the early stages of the exposure, limiting the recording of high-resolution information (Vinothkumar *et al.*, 2014 ▸). The last frames were excluded in order to limit the accumulated exposure to less than 30 e^−^ Å^−2^ and therefore to reduce the loss of high-resolution information owing to radiation damage. The stringent selection of images for processing, based on the recovery of isotropic high-resolution information in their power spectra as described above, and the fact that for each of those images the selected frames were effectively acquired within 0.45 s, with negligible effects owing to microscope-stage drift, led us to judge that alignment of the frames recorded for each exposure was not required. For both the human and the *P. falciparum* 20S proteasome samples, all images were recorded from a single cryo-EM grid during a single data-collection session.

## 3. Image-processing strategy   

The single-particle analysis refinement routines used in the processing of the data sets for both the human and the *P. falciparum* 20S proteasomes have been described, together with the strategy for protein model building (da Fonseca & Morris, 2015 ▸; Li, O’Donoghue *et al.*, 2016 ▸). Briefly, the single-particle analysis refinement routines consisted of rounds of image alignment and angular assignment by projection matching using the *AP SH* program from the *Spider* software package (Frank *et al.*, 1996 ▸), and three-dimensional reconstruction and three-dimensional forward projections using the locally developed programs *icr*3*d* (Institute of Cancer Research 3D reconstruction) and *icr*3*dpro* (Institute of Cancer Research 3D projections), respectively. The programs *icr*3*d* and *icr*3*dpro* are described in detail below. In the analysis of both the human and the *P. falciparum* 20S proteasomes, a crucial step was the use of an appropriate initial reference. For this purpose, we originally used a model map, low-pass filtered to 20 Å, calculated from coordinates fitted into the 20S core region of a cryo-EM map of the human 26S proteasome (da Fonseca *et al.*, 2012 ▸). This filtered model map was found to retain sufficient detail to still allow differentiation between the closely related seven α and seven β subunits, which is required to avoid incorrect alignment to reference projections related by pseudo-sevenfold symmetry, while at the same time avoiding model bias. Initially, this reference was used as the starting model for the analysis of the human 20S proteasome core in the apo state (unpublished data). The resulting map, low-pass filtered to 20 Å, was used as the starting reference for the analysis of a ligand-bound human proteasome (da Fonseca & Morris, 2015 ▸), which in turn was used as the starting reference for the analysis of the *P. falciparum* complex (Li, O’Donoghue *et al.*, 2016 ▸).

### 3.1. The *icr*3*d* and *icr*3*dpro* programs   

#### 3.1.1. Geometrical weighting   

**Figure 2:**
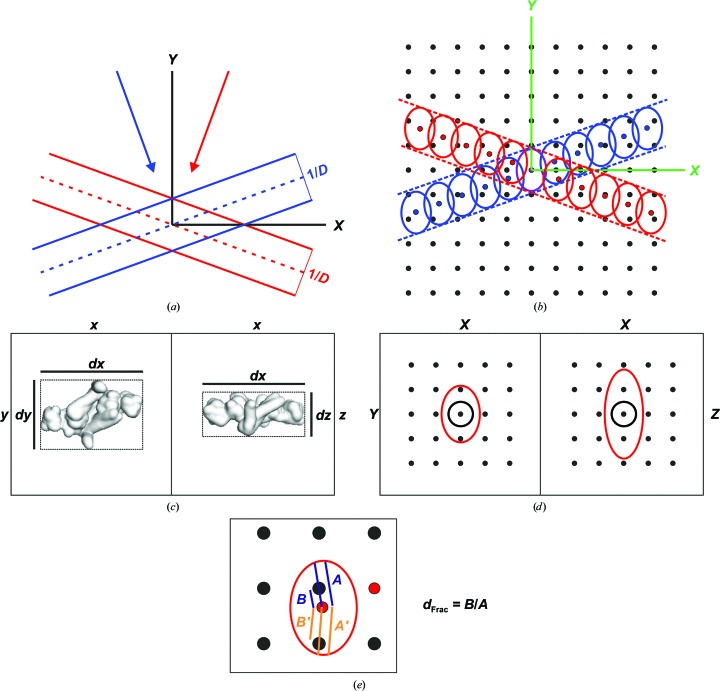
Scheme for the Fourier-space reconstruction used in the program *icr*3*d*. (*a*) Three-dimensional reconstruction in Fourier space by the summation of central sections (two are shown, one in blue and one in red), which derive from the projection angles of the two-dimensional images (indicated by red and blue arrows). The depth of each central section depends on the reciprocal dimension of the reconstructed object (1/*D*) measured in the projection direction. (*b*) Contributions from the input Fourier components of two individual particle images (red and blue points) to the output Fourier components of the three-dimensional reconstruction (black points) are confined to the central section (red and blue dashed lines) as in (*a*). These are added to neighbouring three-dimensional Fourier components within ellipsoidal contributing envelopes (red and blue ellipses), the dimensions of which are reciprocally related to the maximum dimensions of the reconstructed object in the relevant directions. (*c*) Maximum dimensions of a reconstructed object expressed as fractions of the cubic reconstructed volume shown in the *xy* and *xz* planes. (*d*) Corresponding ellipsoidal Fourier-space contribution envelopes (red ellipses), which can be compared with the spherical contribution envelopes (black circles) that relate to a reconstructed object with maximum dimensions equal to the cubic reconstructed volume. (*e*) The fractional distance (*d*_Frac_) used to determine sinc-weighted contributions to the output Fourier components is calculated from the distances (*B* and *B*′) between the input Fourier components (red dots) and the output Fourier components (black dots) as a fraction of the distance to the edge of the ellipsoidal contributing envelope (*A* and *A*′).

For our analysis of both the human and *P. falciparum* 20S proteasomes, three-dimensional maps were calculated using a locally developed program, *icr*3*d*, while a second closely related program, *icr*3*dpro*, was used to generate two-dimensional reprojections from these three-dimensional maps, which were used for refinement of the alignment and angular assignment parameters of the data. In single-particle analysis, three-dimensional reconstructions from projection images are commonly calculated by either real-space or Fourier-space methods (Grigorieff, 2007 ▸). Real-space reconstructions typically involve weighted back-projection algorithms. Here, individual two-dimensional images are back-projected into a three-dimensional volume in directions corresponding to their projection directions (characterized by Euler angles), which have previously been estimated by projection matching or angular reconstitution. The reconstructed volume is the sum of these back-projected contributions. Consequently, it is necessary to use a weighting procedure to compensate for the unequal contributions at different spatial resolutions resulting from this approach. The exact-filter three-dimensional reconstruction algorithm (Harauz & van Heel, 1986 ▸) performs this function, as well as taking into account the uneven angular distributions in the input data that are often encountered in cryo-EM experiments. The exact filter exploits the equivalence between two-dimensional projections of a three-dimensional object and central sections of its three-dimensional Fourier transform. These central sections have a depth that is reciprocally related to the dimension of the reconstructed object in the projection direction. The finite depth of the central sections leads to Fourier contributions from different images overlapping (Fig. 2 ▸ *a*), and the extent of overlap can be used to obtain Fourier-space weighting functions, which serve to attenuate such regions of multiple overlapping contributions. The weighting functions are used to filter Fourier transforms of the input images, which are then back-transformed into real space and back-projected to give the three-dimensional reconstruction.

In the *icr*3*d* program an equivalent weighting approach to that of Harauz & van Heel (1986 ▸) has been implemented, but in this case the input image data are merged in Fourier space. Fourier transforms of the input data are subjected to coordinate transformation defined by their assigned Euler angles to create central sections through the three-dimensional Fourier transform of the reconstructed volume. At this stage, the rotational and translational parameters obtained during the alignment of the individual molecular images against the current reference structure can be applied to original un­interpolated images by phase-shifting the Fourier components, thereby minimizing the number of interpolations in deriving the three-dimensional structure. Contributions from each Fourier component of the input images are added to those Fourier coefficients of the output three-dimensional Fourier transform that fall within a contribution envelope (Fig. 2 ▸ *b*). The dimensions of the contribution envelope are calculated in a similar way to the depths of the Fourier-space central sections in the exact-filter back-projection approach, *i.e* 1/*D* (Figs. 2 ▸ *a* and 2 ▸ *b*), where *D* is the linear dimension of the object (van Heel & Harauz, 1986 ▸). Given that the generalized object may have different linear dimensions along each of its major axes (Fig. 2 ▸ *c*), the Fourier-space contribution envelope exists as an ellipsoid and the dimensions of its axes correspond to half the reciprocal dimension of a bounding cuboid containing the reconstructed object (Figs. 2 ▸ *c* and 2 ▸ *d*). Each Fourier component of the input image will contribute to the Fourier components of the reconstructed object that lie within its contribution envelope (Figs. 2 ▸ *b* and 2 ▸ *e*), and each Fourier component of the reconstructed object is calculated as a weighted average of all of its contributing input image Fourier components. Individual contributing Fourier components are weighted by a geometrical weighting factor (*W*_geom_) evaluated as a sinc function of the fractional distance between the input Fourier component and the output Fourier component to which it is contributing, *d*_Frac_ (Fig. 2 ▸ *e*), 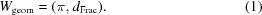



The resulting output three-dimensional Fourier components correspond to the geometrically weighted average of input Fourier components using a Wiener filter to avoid excessive noise being introduced into Fourier components where there are a small number of contributions from the input data (Grigorieff, 2007 ▸),
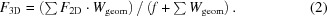



The weighting approach adopted here is similar to that used in the three-dimensional analysis of two-dimensional crystals, where sinc functions are fitted along reciprocal-lattice lines through irregularly distributed sets of Fourier components from tilted images to provide the Fourier components of a regular grid required for three-dimensional Fourier synthesis (Amos *et al.*, 1982 ▸). This provides an effective means of interpolation between the non-integral sample points arising from the two-dimensional input data and the output integral three-dimensional grid. Furthermore, by setting the contribution envelope to match the dimensions of the reconstructed object, more averaging can be achieved, potentially improving the signal-to-noise ratio. This geometrical weighting approach is the major novel feature of *icr*3*d*.

Finally, in *icr*3*d* the Fourier transforms of both the input images and the three-dimensional reconstruction can be subsampled, in order to increase the reconstruction accuracy, by padding the real-space input images in boxes *n* times the original dimensions. Typical values for *n* are 2 or 3, depending on the image box size. Both our human and *P. falciparum* 20S proteasome cryo-EM maps were calculated using a padding factor of 3.

#### 3.1.2. The contrast-transfer function   

In *icr*3*d*, the contrast-transfer function (CTF) correction is achieved in two steps. Firstly, correction of the phases is carried out by phase reversal in the appropriate frequency zones in the Fourier transforms of complete microscope images prior to particle selection, thereby maximizing the recovery of information from individual images delocalized by the point-spread function into neighbouring regions. In our analysis of the 20S proteasome, this was performed with the *Tigris* program *flipctf* using the defocus values calculated using the *Tigris* program *findctf*. Conversely, correction for the amplitude oscillations arising from the CTF is more effectively achieved at the stage of the weighted merging of the Fourier components from individual molecular images into the reconstructed three-dimensional Fourier transform. This avoids boosting the noise in the frequency zones where the CTF is close to zero. Additional weighting terms for the input images are included in *icr*3*d* to take account of a defocus-dependent envelope value (*W*_def_) for each Fourier component, together with the correlation coefficient for the whole input image. In each case, these terms serve to upweight Fourier components where the signal-to-noise ratio is higher. Combined, this gives rise to a global weighting function (*W*_global_),
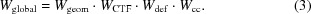



Accordingly, three-dimensional Fourier components are obtained as follows:
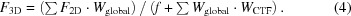



The parameters used for the defocus envelope weighting function result in the sharpening of the reconstruction, and therefore optimal sharpening of the resulting map can be achieved by applying relatively small values of negative *B* factor. For the interpretation of both the human and the *P. falciparum* cryo-EM maps, a *B* factor of −50 Å^2^ was used to aid in model building (da Fonseca & Morris, 2015 ▸; Li, O’Donoghue *et al.*, 2016 ▸).

#### 3.1.3. The *icr*3*d* program and its input parameters   

**Figure 3:**
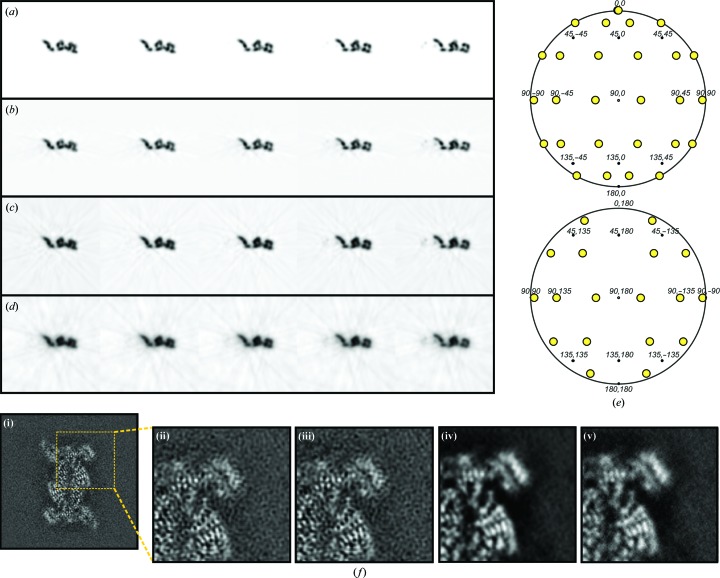
Effect of the different parameters used in *icr*3*d* reconstructions. (*a*–*e*) Tests with the model density illustrated in Fig. 2 ▸(*c*). (*a*) Sections of model density. (*b*, *c*, *d*) Sections from reconstructed density produced from input projection images sampled at 30° intervals. (*b*) Sections from reconstruction with the contribution envelope set to match the dimensions of the reconstructed object as illustrated in Fig. 2 ▸(*c*) (*i.e.* the standard mode of usage for *icr*3*d*). (*c*) Sections from reconstruction with the contribution envelope set to match the dimensions of the reconstructed volume. (*d*) Sections from reconstruction calculated with the contribution envelope set to match the dimensions of a reconstructed volume of twice the size used in (*c*). (*e*) Distribution of Euler angles of the input projection images with 30° sampling, used to calculate the maps in (*b*–*d*). (*f*) Reconstruction of the human 20S proteasome from cryo-EM data (da Fonseca & Morris, 2015 ▸) illustrating the effect of different parameters on a single section of reconstructed density. (i) Reconstruction using CTF weighting, CTF amplitude correction and set to match the dimensions of the 20S proteasome; (ii) magnified region of image (i); (iii) as (ii) but with the contribution envelope set to match the reconstructed volume; (iv) as (ii) but with no CTF amplitude correction; (v) as (iv) but with no CTF weighting.

Once all of the input data have been merged in Fourier space, as described above, three-dimensional reconstructions in real space are obtained by Fourier transformation. With an adequate choice of input parameters, the resulting reconstructions obtained using *icr*3*d* are characterized by a high signal-to-noise ratio, with unambiguous protein densities that are clearly distinguishable from the background, while retaining the recovery of high-resolution details (Fig. 3 ▸), as observed in our maps of the human and the *Plasmodium* 20S proteasomes (da Fonseca & Morris, 2015 ▸; Li, O’Donoghue *et al.*, 2016 ▸).

Figs. 3 ▸(*a*)–3 ▸(*e*) illustrate the effects of geometrical weighting and varying the contribution envelope of each input Fourier component on three-dimensional reconstructions. These were calculated using a test data set obtained by forward-projecting the model map shown in Fig. 2 ▸(*c*), which is characterized by an axial ratio of 2.3, using a sparse set of projection images. Using *icrd*3*d* with the contribution envelope set to match the linear dimensions of the reconstructed object results in good recovery of the internal detail of the reconstruction (Fig. 3 ▸ *b*) compared with the original model map (Fig. 3 ▸ *a*). The background is quite clean, although with some residual modulation arising from the deliberate use of projection images with Euler angles with 30° spacing (Fig. 3 ▸ *e*). Conversely, significant internal detail is lost coupled to the exaggeration of low frequencies and increased background noise if the contribution envelope is set to match the dimensions of the reconstructed volume (Fig. 3 ▸ *c*). These effects are further exaggerated if the contribution envelope dimensions are set to match a reconstructed volume which is doubled in size (Fig. 3 ▸ *d*). This overrepresentation of low-frequency information has previously been observed with the related exact-filter back-projection approach applied to filamentous systems which have inherently high axial ratios (Paul *et al.*, 2004 ▸). The effect of the additional weighting parameters can be assessed with experimental cryo-EM images of the human 20S proteasome (da Fonseca & Morris, 2015 ▸; Fig. 3 ▸ *f*). Here, the reconstruction calculated with optimized weighting factors (Fig. 3 ▸ *f*, i and ii) is compared with reconstructions with no allowance for the dimensions of the reconstructed object (Fig. 3 ▸ *f*, iii), without CTF amplitude correction (Fig. 3 ▸ *f*, iv) and without CTF weighting (Fig. 3 ▸ *f*, v). In each case the signal-to-noise ratio is reduced, with an additional attenuation of high frequencies when the reconstructions are calculated using no amplitude correction or CTF weighting (Fig. 3 ▸ *f*, iv and v).

The programs *icr*3*d* and *icr*3d*pro* are written in C++ as part of the *Tigris* package. *icr*3*d* reads input images and their Euler angles in *IMAGIC* format (van Heel *et al.*, 1996 ▸) and outputs a three-dimensional reconstruction also in *IMAGIC* format. *icr*3*dpro* takes a three-dimensional density map and a list of Euler angles and returns a set of two-dimensional projections using the geometrical weighting approach. Both programs are implemented in the *Tigris* software package, which is publicly available at https://sourceforge.net/projects/tigris/.

## 4. The cryo-EM structure of a ligand-bound human 20S proteasome: a proof of principle   

**Figure 4:**
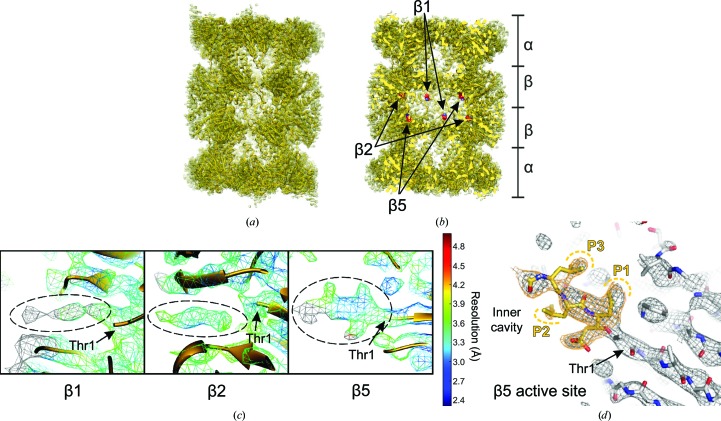
Cryo-EM structure of the human 20S proteasome with an inhibitor bound, showing the map and coordinates deposited in the Electron Microscopy Data Bank and Protein Data Bank with accession codes EMD-2981 and 5a0q, respectively (da Fonseca & Morris, 2015 ▸). (*a*) Overall view of the density map (mesh) and protein coordinates (cartoon). (*b*) The map shown in (*a*) cut to reveal the location in the proteasome inner cavity of the Thr1 residues (shown as spheres) at the active sites of subunits β1, β2 and β5. (*c*) Close-up views of the three active sites, showing the map densities (mesh representation) colour-coded according to the local resolution estimated with *ResMap* (Kucukelbir *et al.*, 2014 ▸) with protein coordinates represented as a cartoon. Densities for the ligand (encircled by dashed lines) are clearly seen extending from the Thr1 residues of subunits β1, β2 and β5 (indicated by arrows). (*d*) Close-up view of the cryo-EM map (mesh representation) and model coordinates (shown as sticks) for the β5 subunit of the human 20S proteasome, with the L_3_VS moiety of the inhibitor (coloured yellow) extending from the Thr1 residue (indicated by an arrow). The ligand LLL tripeptide mimics proteasome substrate positions P1–P3, as labelled, where the side chains at positions P1 and P3 are oriented towards the ligand-binding pocket, while that at position P2 is oriented towards the inner cavity of the proteasome. (*a*), (*b*) and (*c*) were created using *UCSF Chimera* (Pettersen *et al.*, 2004 ▸) and (*d*) was created using *PyMOL* (Schrödinger).

X-ray crystallography has been extensively used to study the structural details of the interaction of eukaryotic 20S proteasomes with inhibitory ligands, particularly using complexes purified from *S cerevisiae*, in order to guide drug discovery. Building on this, we investigated the suitability of using cryo-EM for such studies, taking advantage of the recent advances in the field, using human 20S proteasomes with a ligand bound (da Fonseca & Morris, 2015 ▸). The chosen ligand was adamantaneacetyl-(6-aminohexanoyl)_3_-(leucyl)_3_-vinylmethyl-sulfone (AdaAhx_3_L_3_VS), a highly potent proteasome inhibitor that covalently binds to the Thr1 residue of the proteolytically active subunits of the 20S proteasome (Bogyo *et al.*, 1997 ▸; Kessler *et al.*, 2001 ▸). In our cryo-EM map (Figs. 4 ▸ *a* and 4 ▸ *b*), the protein backbone of each individual proteasome subunit is clearly identified and densities are resolved for most of the proteasome side chains, consistent with the estimated resolution of about 3.5 Å. Furthermore, extra densities extending from the proteolytically active Thr1 residues of the β1, β2 and β5 subunits (Fig. 4 ▸ *b*) can be directly assigned to the L_3_VS moiety of the ligand AdaAhx_3_L_3_VS (Figs. 4 ▸ *c* and 4 ▸ *d*). No densities were recovered for the AdaAhx_3_ moiety of the ligand at any of the three proteasome active sites, which is consistent with a flexible conformation of this part of the ligand within the proteasome inner cavity.

In the cryo-EM map of the human 20S–AdaAhx_3_L_3_VS complex (da Fonseca & Morris, 2015 ▸), the L_3_VS moiety is particularly well resolved in the β5 active site, clearly showing its extended near-planar conformation and allowing molecular building of the vinyl-sulfone group and the three leucine side chains (Figs. 4 ▸ *c* and 4 ▸ *d*). The densities for the L_3_VS moiety of the ligand at the β2 and β1 active sites are weaker than those extending from the β5 Thr1 (Fig. 4 ▸ *c*), which can be attributed to lower ligand occupancy at these active sites. This is consistent with the results obtained from *in vitro* assays of the inhibition of mammalian proteasomes by AdaAhx_3_L_3_VS, which show higher potency of the ligand towards the β5 active site than to those in the β1 and β2 subunits (Kessler *et al.*, 2001 ▸). The consistency of proteasome active-site occupancy observed in our cryo-EM map and the ligand potency towards the different proteasome active sites is a consequence of the use of similar close-to-physiological conditions, and illustrates one of the main advantages of using cryo-EM in the study of protein–ligand interactions in general (da Fonseca & Morris, 2015 ▸). The preservation of such optimal ligand-binding conditions is commonly compromised when studying protein–ligand interactions by X-ray crystallography, which has been the method of choice for the study of eukaryotic 20S proteasomes. In these studies, pre-formed protein crystals are soaked in solutions containing high ligand concentrations, under conditions that primarily must preserve the integrity of the protein crystals rather than mimicking those for physiological protein–ligand interactions. Under such conditions ligand selectivity can be difficult to be preserve, particularly when comparing closely related ligands and/or when targeting closely related active sites.

Apart from allowing the structural study of protein–ligand interactions under conditions that are closer to physiological, cryo-EM also has the important advantage of requiring significantly lower amounts of protein than other methods of structure determination. This extends the feasibility of high-resolution structural analysis to protein samples that, for biochemical reasons, are difficult to prepare in high quantities. An example is the 20S proteasome from *P. falciparum*, our cryo-EM structure of which is now being explored in the fight against malaria (Li, O’Donoghue *et al.*, 2016 ▸; Li, Bogyo *et al.*, 2016 ▸), as outlined below.

## 5. The cryo-EM structure of the *P. falciparum* proteasome in the discovery of new antimalarials   

*P. falciparum* is the parasite that is responsible for the most severe form of malaria. This mosquito-transmitted disease affects hundreds of millions of people every year, particularly in tropical and subtropical climates. According to the World Health Organization, the vast majority of the hundreds of thousands of people killed by malaria in 2015 were young children under the age of five (World Health Organization, 2015 ▸). Artemisinin derivatives are now the front-line antimalarials, and while the number of people affected by malaria is still staggering, the use of artemisin-based combination therapies has contributed to a recent significant reduction in the world malaria burden. However, resistance of *P. falciparum* to artemisinin derivatives has emerged in Southeast Asia (Ashley *et al.*, 2014 ▸; Tilley *et al.*, 2016 ▸), and its spread represents a serious threat to human health and to the current efforts towards the global control and eventual eradication of malaria, urging the development of new efficient antimalarials. Inhibition of the *Plasmodium* proteasome is toxic to the parasite at all stages of its life cycle (Gantt *et al.*, 1998 ▸), and it has been suggested that the *Plasmodium* proteasome can be specifically targeted (Li *et al.*, 2012 ▸; Li, Tsu *et al.*, 2014 ▸; Li, van der Linden *et al.*, 2014 ▸). These studies indicate a potential role of parasite proteasome inhibition in the development of next-generation antimalarials. However, the development of proteasome inhibitors as antimalarials requires knowledge, at the molecular level, of the differences between the parasite and human proteasome ligand-binding preferences, in order to guide the development of highly specific drugs with thera­peutic potential.

High-resolution structural information is required in order to fully understand the molecular basis of ligand specificity and in order to serve as a framework for the development of specific drugs with therapeutic potential. While X-ray crystallography has been the method of choice for the structural analysis of 20S proteasome–ligand interactions, the low yield of 20S proteasome samples obtained from *P. falciparum* cultures makes its crystallization impractical. On the other hand, the preparation of cryo-EM grids requires a significantly lower amount of protein than crystallization. A cryo-EM grid can be prepared using as little as 2 µl of sample at a protein concentration of about 0.1 mg ml^−1^, when using electron-microscope grids coated with a continuous thin layer of carbon, although batches of grids must be prepared for the optimization of freezing conditions and cryo-EM data collection. Hence, we built on our previous experience with the cryo-EM analysis of the human 20S proteasome with a ligand bound (da Fonseca & Morris, 2015 ▸) in order to determine the structure of the *P. falciparum* proteasome (Li, O’Donoghue *et al.*, 2016 ▸).

**Figure 5:**
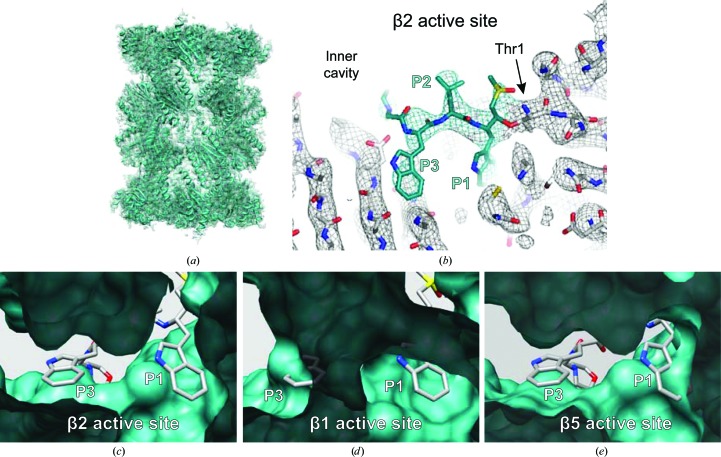
Cryo-EM structure of the *P. falciparum* 20S proteasome with an inhibitor bound, showing the map and coordinates deposited in the Electron Microscopy Data Bank and Protein Data Bank with accession codes EMD-3231 and 5fmg, respectively (Li, O’Donoghue *et al.*, 2016 ▸). (*a*) Overall view of the density map (mesh) and protein coordinates (cartoon). (*b*) Close-up view of the density map (mesh) and model coordinates (sticks) showing the WLW-vs inhibitor (teal) bound to Thr1 of the β2 subunit. The ligand WLW tripeptide mimics proteasome substrate positions P1–P3, as labelled. As for other tripeptide proteasome inhibitors, the side chains at positions P1 and P3 are oriented towards the ligand-binding pocket, while that at position P2 is oriented towards the inner cavity of the proteasome. (*c*) Model of the *P. falciparum* β2 active site with bound ligand, as determined by cryo-EM, viewed towards the inner cavity of the proteasome. (*d*) *P. falciparum* β1 active site with superimposed inhibitor coordinates, showing that the tryptophan side chains of the ligand, at positions P1 and P3, cannot be accommodated at this active site owing to steric constraints. (*e*) *P. falciparum* β5 active site with superimposed inhibitor coordinates, showing that the tryptophan side chain of the ligand at position P1 cannot be accommodated in this active site owing to steric constraints. In (*c*), (*d*) and (*e*) the protein model is represented as van der Waals surfaces and the ligand as sticks. (*a*), (*c*), (*d*) and (*e*) were created using *UCSF Chimera* (Pettersen *et al.*, 2004 ▸) and (*b*) was created using *PyMOL* (Schrödinger).

The high-resolution cryo-EM structure of the *P. falciparum* 20S proteasome (Fig. 5 ▸) was determined with the complex bound to a new prototype specific inhibitor, WLW-vs, that was identified by extensive biochemical and functional assays (Li, O’Donoghue *et al.*, 2016 ▸). This compound is a peptide-vinyl sulfone that, like other standard proteasome inhibitors, comprises a tripeptide moiety (WLW) that mimics the proteasome substrate positions P1–P3, counted upstream from the proteolytic scissile bond. The side chains of these three amino-acid residues confer specificity towards the different proteasome proteolytic sites. Functional assays revealed that this compound is unusual in its binding preference towards the β2 subunit of the *Plasmodium* proteasome, while avoiding binding to the parasite proteasome β1 and β5 active sites and all of those in the human complex (Li, O’Donoghue *et al.*, 2016 ▸). This specificity is confirmed by our cryo-EM structure of the *Plasmodium* 20S–WLW-vs complex at a resolution of about 3.6 Å, where the ligand is found only at the *Plasmodium* β2 binding pocket (Fig. 5 ▸ *b*). Our structure clearly shows that the molecular basis for this unusual selectivity arises from the unpredictably spacious *Plasmodium* β2 binding pocket, which permits accommodation of the ligand tryptophan side chains (Fig. 5 ▸ *c*), while binding to the *Plasmodium* β1 and β5 pockets is shown to be impaired by steric constraints (Figs. 5 ▸ *d* and 5 ▸ *e*). Steric constraints also impair the binding of WLW-vs to all of the human proteasome active sites, thus explaining the specificity of this compound towards the parasite complex, as was shown previously (Li, O’Donoghue *et al.*, 2016 ▸). Most importantly, our structure provides a suitable framework to assist the drug development currently in progress for the improvement of *Plasmodium* proteasome specific inhibitors into potential antimalarials for clinical use (Li, Bogyo *et al.*, 2016 ▸).

## 6. Future prospects   

Here, we have described in detail the methods that we have used to determine the cryo-EM structures of ligand-bound human and *P. falciparum* 20S proteasomes, namely the strategy to avoid orientation bias of the human proteasome on cryo-EM grids and our approaches for data collection and image analysis, with emphasis on the details of the *icr*3*d* three-dimensional reconstitution algorithm. Our studies demonstrate the feasibility and advantages of using cryo-EM and single-particle analysis to derive the structures of ligand-bound protein complexes relevant to guide the design and improvement of effective drugs for clinical use (da Fonseca & Morris, 2015 ▸; Li, O’Donoghue *et al.*, 2016 ▸; Li, Bogyo *et al.*, 2016 ▸). These studies have established and exploited the ability of cryo-EM to allow work under near-physiological solution conditions and inhibitor concentrations similar to those used for *in vitro* ligand-binding assays, which are usually not attainable using other methods of protein structure determination. Consequently, cryo-EM permits the structural analysis of protein–ligand complexes where binding selectivity is preserved, and where ligand occupancy is found to be consistent with the results of *in vitro* binding assays. This is of particular relevance when investigating the structural basis for ligand selectivity between chemically related ligands and/or active sites. In the eukaryotic proteasome, the substrate selectivity of the three related but distinct active sites associated with the β_1_, β_2_ and β_5_ subunits is dictated by constraints resulting from the different amino-acid side chains lining each of the three substrate-binding pockets (Fig. 5 ▸). The existence of three closely related proteolytic active sites with distinct substrate preferences within the eukaryotic 20S proteasome makes it a particularly interesting system to investigate by cryo-EM, with the potential to provide information suitable to understand the structural basis for their selectivity.

Our work shows that cryo-EM can be used to resolve not only ligand selectivity between the three distinct proteasome active sites, as illustrated in Fig. 5 ▸, but also ligand-binding specificity between the human and *Plasmodium* complexes, as we have previously reported (da Fonseca & Morris, 2015 ▸; Li, O’Donoghue *et al.*, 2016 ▸). This type of approach can in principle be extended to the study and optimization of the selective inhibition of different classes of human proteasomes (constitutive proteasomes, immunoproteasomes or thymoproteasomes) for potential therapeutic usage. Furthermore, we showed that although they are closely related there are differences between the human and *P. falciparum* 20S proteasome active sites that allow specific targeting in the development of potential new antimalarials, and it is likely that the same applies to other disease-causing protozoan parasites. As for the analysis of the *P. falciparum* proteasome, here cryo-EM structures may be valuable owing to both the requirement for smaller amounts of protein and the preservation of ligand selectivity (Li, Bogyo *et al.*, 2016 ▸; Bibo-Verdugo *et al.*, 2017 ▸). In this context, a new compound was recently identified that selectively targets 20S proteasomes from pathogenic kinetoplastid parasites, namely *Trypanosoma cruzi*, *T. brucei* spp. and *Leishmania* spp., which cause Chagas disease, sleeping sickness and leishmaniasis, respectively (Khare *et al.*, 2016 ▸). This new compound does not inhibit mammalian proteasomes and acts by a noncompetitive mechanism, although a direct structural analysis is still required to fully characterize its inhibitory mechanisms at the molecular level.

We have focused on our cryo-EM structural studies of ligand-bound eukaryotic 20S proteasomes and how these have identified advantages in using cryo-EM to study ligand-binding interactions, aiming at the development of new improved therapeutic drugs, in particular antimalarials. More generally, the recent advances in the cryo-EM field have changed the overall perception of its use for the detailed study of intricate protein structures, which has been accompanied by an increased interest in its application in drug discovery and development (Subramaniam *et al.*, 2016 ▸; Merino & Raunser, 2017 ▸). While in our studies of eukaryotic 20S proteasomes we used ligands that covalently bind specific proteolytic active sites, the utility of cryo-EM to also study noncovalent protein–ligand interactions has been shown by others (Merk *et al.*, 2016 ▸). Examples of other high-resolution cryo-EM structures with resolved densities for exogenous ligands include those of ribosomes (Wong *et al.*, 2014 ▸; Fischer *et al.*, 2015 ▸; Myasnikov *et al.*, 2016 ▸), TRPV1 (Gao *et al.*, 2016 ▸), p97 (Banerjee *et al.*, 2016 ▸) and lactate dehydrogenase (Merk *et al.*, 2016 ▸). While cryo-EM does not yet have the high throughput of other structural biology methods, its advantages in the study of protein structures and protein–ligand interactions under near-physiological conditions have been demonstrated. These, together with the rapid ongoing advances in cryo-EM instrumentation and image-analysis tools, which are both extending the use of cryo-EM to study wider ranges of protein and protein–ligand complexes and increasing the high resolutions attainable (Vinothkumar & Henderson, 2016 ▸), clearly indicate that cryo-EM will play an increasingly relevant role in structural biology and in the development of new and improved therapeutic drugs.
